# Real-world rogue wave probabilities

**DOI:** 10.1038/s41598-021-89359-1

**Published:** 2021-05-12

**Authors:** Dion Häfner, Johannes Gemmrich, Markus Jochum

**Affiliations:** 1 Niels Bohr Institute, University of Copenhagen, Copenhagen, Denmark; 2 University of Victoria, Victoria, BC Canada

**Keywords:** Ocean sciences, Natural hazards, Scientific data

## Abstract

Rogue waves are dangerous ocean waves at least twice as high as the surrounding waves. Despite an abundance of studies conducting simulations or wave tank experiments, there is so far no reliable forecast for them. In this study, we use data mining and interpretable machine learning to analyze large amounts of *observational data* instead (more than 1 billion waves). This reveals how rogue wave occurrence depends on the sea state. We find that traditionally favored parameters such as surface elevation kurtosis, steepness, and Benjamin–Feir index are weak predictors for real-world rogue wave risk. In the studied regime, kurtosis is only informative within a single wave group, and is *not* useful for forecasting. Instead, crest-trough correlation is the dominating parameter in all studied conditions, water depths, and locations, explaining about a factor of 10 in rogue wave risk variation. For rogue crests, where bandwidth effects are unimportant, we find that skewness, steepness, and Ursell number are the strongest predictors, in line with second-order theory. Our results suggest that linear superposition in bandwidth-limited seas is the main pathway to “everyday” rogue waves, with nonlinear contributions providing a minor correction. This casts some doubt whether the common rogue wave definition as any wave exceeding a certain height threshold is meaningful in practice.

## Introduction

An extreme ocean wave (“rogue wave” or “freak wave”) is commonly defined as any wave that is higher than 2 or 2.2 times the significant wave height $H_S$, and they pose a substantial threat to seafaring vessels and offshore structures^1^.

Despite having been in research focus for almost 25 years, they are still being studied extensively^2–7^. By now, we know several ways to produce truly exceptional waves in wave tanks and simulations^8–10^. However, things are more difficult in the real ocean, where theoretical assumptions (such as unidirectionality) break down. The causes of real-world rogue waves are therefore still unknown, and heavily debated^11–18^.

In recent years, more and more studies approached the problem from a different angle: by inferring the dependence of rogue wave occurrence on the sea state from observed field data^3,5,11,18^. However, no study has so far quantified the probability to encounter a rogue wave depending on the sea state throughout a wide regime of conditions, taking into account more than one parameter at a time, and in a statistically robust fashion. Here, we aim to fill this gap.

In this study, we use FOWD (the Free Ocean Wave Dataset)^19^, a wave catalogue based on data recorded by buoys in 158 different locations around the US coasts and overseas territories, based on raw data from CDIP^20^ (Coastal Data Information Program). We use the pre-filtered version of FOWD-CDIP (v0.4.4) containing about 1.5 billion individual waves (of which about 100,000 exceed $2H_S$), which has already removed faulty deployments and waves recorded during conditions where buoys are unreliable.

We create an aggregated version of the full dataset that bundles together 100 waves at a time (see “Methods”), and are thus able to analyze all sea states simultaneously using robust Bayesian statistics and machine learning. By finding the conditions that show the highest rogue wave probability, we aim to test some common hypotheses concerning rogue waves and their creation mechanisms. To this end, we include only a subset of 12 sea state parameters that we can meaningfully tie to a (hypothesized) cause of rogue waves or crests (Table 1).

**Table 1:** The sea state parameters examined in this study.

Parameter	Physical meaning	References
Crest-trough correlation	Correlation coefficient between wave crest heights and trough depths	^18,21,22^
Spectral bandwidth	Spectral peak width, controls wave group dynamics	^11^
Mean period	Mean wave period	^2^
Rel. low-frequency energy	Relative low-frequency (swell) energy content	^2,4,23,24^
Directional spread	Short-crestedness of waves	^6^
Ursell number ($\log _{10}$)	Non-linear shallow water effects	^25^
Benjamin–Feir index	Degree of non-linearity, modulational instability	^26–28^
Excess kurtosis	Proneness to outliers of sea surface elevation	^13,26,29^
Steepness	Weakly nonlinear corrections, wave breaking	^15,17^
Significant wave height	Reference wave height, total energy	^14^
Skewness	Shape asymmetry between wave crests and troughs	^13,30^
Relative depth ($\log _{10}$)	Shallow-water effects	^27^

See Table 2 for more information about the estimation of each parameter.

We identify the key control parameters for real-world rogue wave risk via careful examination of the correlation between these parameters and measured rogue wave occurrences. Because many of the parameters are also correlated with each other, we have to account for possible confounding along every step (correlation matrix shown in Supplementary Figure S1).

The upcoming sections present the results of this analysis, followed by a discussion of possible limitations and conclusive remarks.

## Results

Throughout the following sections, we characterize the extremeness of a wave or crest by its abnormality index ($\mathrm {AI}$ for waves and $\mathrm {CAI}$ for crests). This is defined as $\mathrm {AI}= H / H_S$ and $\mathrm {CAI}= \eta / H_S$, where *H* is the measured zero-crossing wave height, $\eta$ the measured crest height, and $H_S$ the 30 min spectral significant wave height.

Unless stated otherwise, all analysis is based on the full, aggregated FOWD-CDIP dataset (or stratified versions of it).

The following sections present the 4 main results of this study.

### Bandwidth effects are the dominant pathway to rogue waves

To quantify how the rogue wave probability *p* depends on the sea state, we first examine how *p* changes when varying one sea state parameter at a time. Here, *p* is defined as the probability of any given wave to exceed the rogue wave threshold, i.e., $p = {\text {Pr}}[\mathrm {AI}> y]$ with $y=2.0$ and, where we have enough data, also $y=2.4$.

We split each sea state parameter *x* (Table 1) evenly into *N* bins, and assume that the associated wave height measurements are independently, identically distributed within each bin (see “Methods”). The “predictive power” ${\mathbb {P}}_x$ of a parameter *x* then quantifies the logarithmic ratio between the highest and lowest binned value of *p*(*x*). For example, a value of ${\mathbb {P}}_x = 2$ implies that *p*(*x*) changes by 2 orders of magnitude as *x* is varied.

**Figure 1:**
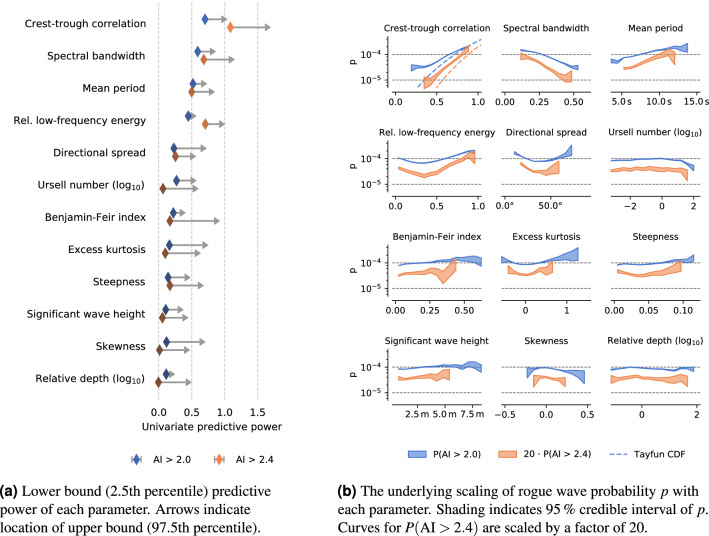
When looking at one sea state parameter at a time, some are better predictors for rogue wave occurrence than others. In particular, crest-trough correlation and spectral bandwidth are much more informative than e.g. Benjamin–Feir index and steepness. (**a**) Shows the predictive power of each parameter, which is computed from the range spanned by the curves in (**b**) (the variation of the rogue wave probability with each parameter).

Applying this binning, we find that crest-trough correlation has the highest univariate predictive power out of all parameters (Fig. 1), explaining about 1 order of magnitude in variation of *p* (with values ranging between $3 \cdot 10^{-5}$ and $2 \cdot 10^{-4}$ for $\mathrm {AI}=2$). Spectral bandwidth, mean period, and low-frequency energy content are also informative with ${\mathbb {P}}$ between 0.5 and 0.8, but these parameters are strongly correlated with crest-trough correlation, so we have to control for possible confounding.

To examine whether spectral bandwidth or crest-trough correlation is the real causal factor, we stratify our analysis on each of these parameters. When stratifying on spectral bandwidth, crest-trough correlation is still the most informative parameter with ${\mathbb {P}} \approx 0.5$, while all other parameters drop to ${\mathbb {P}} < 0.2$. When stratifying on crest-trough correlation, all other parameters become unimportant with most values of ${\mathbb {P}}$ between 0 and 0.2, depending on which value of crest-trough correlation we condition on (see also Supplementary Figure S2).

This implies that spectral bandwidth (and most other parameters) act *through* their correlation with crest-trough correlation. This is strong evidence that crest-trough correlation is the key control parameter for rogue waves, with some other factors serving as minor corrections.

When we take the full, multivariate parameter space into account, things are more difficult to analyze, because interactions between parameters could possibly create “hot corners” of elevated rogue wave activity that are not detectable by univariate analysis. To discover whether this is the case, we run a clustering algorithm that identifies rectangular regions in parameter space where we find higher rogue wave probabilities than in any univariate bin (see “Methods” section).

**Figure 2:**
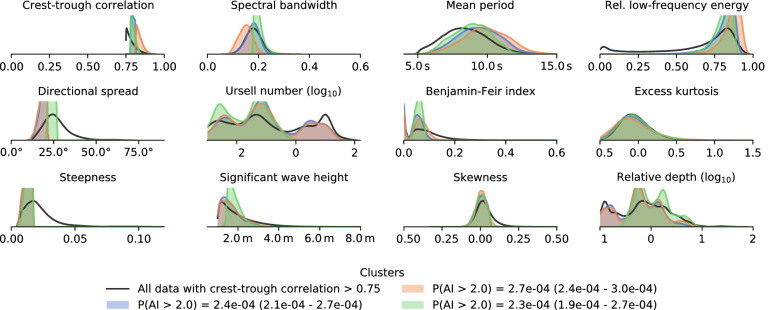
“Hot corners” of rogue wave activity have high crest-trough correlation, strong swells, and low steepness. Shown is the distribution of each cluster population in parameter space, and the distribution of all waves with high crest-trough correlation for comparison. Clusters are computed through decision-tree based clustering (see “Methods”), taking all parameters into account at the same time. All clusters show a higher rogue wave incidence than any univariate bin. Ranges in legend indicate 95% credible interval.

This multivariate analysis reveals that crest-trough correlation is still the most important parameter in all found clusters, where all cluster populations have crest-trough correlations above 0.75 (Fig. 2). All of the clusters are also located in swell-dominated conditions with high mean period, low directional spread, and low steepness. We examine the role of wave period and steepness further below.

### Surface elevation kurtosis does *not* predict rogue waves

The kurtosis (fourth standardized moment) of the sea surface elevation is a commonly studied parameter in connection with rogue waves^13,29,31^, and a central ingredient of ECMWF’s rogue wave forecast^26^. However, some authors have expressed doubt whether a high kurtosis is the *cause* or *effect* of extreme waves^32,33^, as kurtosis is a measure for tail-heaviness of a distribution, and rogue waves are extreme outliers by definition. In other words, we examine the question: is a sea state that is more prone to outliers in the recent past also prone to more outliers (rogue waves) now?

We examine this by studying how the predictive power of kurtosis depends on the time lag between the end of the aggregation period (based on which the sample kurtosis is computed) and the observed wave height. Because we can only study this in non-time aggregated data, which requires 100 times more resources than aggregated data, we need to restrict this analysis to a subset of the full dataset. We use the FOWD data from all Hawaiian CDIP stations (098p1, 106p1, 146p1, 165p1, 187p1, 188p1, 198p1, 225p1, 233p1), containing 160 million waves.

We also include two robust kurtosis estimators in this analysis (based on quantile spread and expected exceedance probabilities^34^), as the sample kurtosis based on the fourth moment of the sea surface elevation is a noisy quantity that is highly sensitive to single extreme measurements. These robust alternatives should be more accurate estimators for the true kurtosis of the sea state (as can be obtained through simulations or very long, controlled experiments under identical conditions).

**Figure 3:**
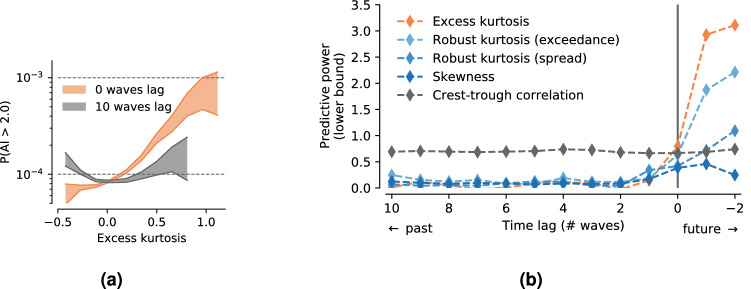
Past sea surface elevation kurtosis is a poor predictor for rogue wave occurrence in the future. Shown is the scaling of the rogue wave probability *p* with kurtosis for 2 different values of time lag (**a**) and the resulting *predictive power* of various quantities depending on time lag (**b**). Here, *time lag* refers to the time between the end of the aggregation period used to compute each sea state parameter and the start of the observed wave.

Results show that even a small time lag of only 3 waves between the end of the aggregation period and observed wave height reduces the predictive power of kurtosis to its (low) background value (Fig. 3). If the kurtosis is computed including future state (negative time lag), it is extremely informative as expected, since rogue wave occurrence *causes* very high values of kurtosis. But even for a time lag of 0, where the end of the aggregation period lies right before the current wave, we discover a substantially elevated predictive power.

We explain this with the common occurrence of multiple rogue waves within the same wave group, where measuring the first rogue wave gives an elevated probability of encountering a second one right after. Indeed, the FOWD dataset contains a relatively high number of multiple rogue waves in rapid succession (about 2500 waves with $\mathrm {AI}> 2$ within 30 s of each other, which corresponds to about 3% of all rogues)^19^.

We also find that the robust kurtosis estimators are not more informative than straightforward sample kurtosis, even though they are indeed less affected by time lag.

We conclude therefore that surface elevation kurtosis is a short-ranged predictor that is only useful within a single wave group, and has little predictive quality otherwise. This has an important implication. If outliers in the past are a poor predictor for outliers in the future, one sensible interpretation is that the encounter of a rogue wave is indeed mostly up to chance (and thus unlikely to elevate the general proneness to outliers in the whole sea state).

### The effects of steepness and Benjamin–Feir index depend on wave period

If we look at how the rogue wave probability depends on spectral energy content (Fig. 1), we notice something curious: *p* attains a local maximum for both very high-frequency and very low-frequency seas. To investigate this, we re-run our analysis for high-frequency and low-frequency conditions.

As low-frequency/high-frequency seas we take all data where the relative energy content in the spectral band 0.05 Hz to 0.1 Hz (representing swell) lies in the interval (0, 0.1) and (0.8, 0.85), respectively.

**Figure 4:**
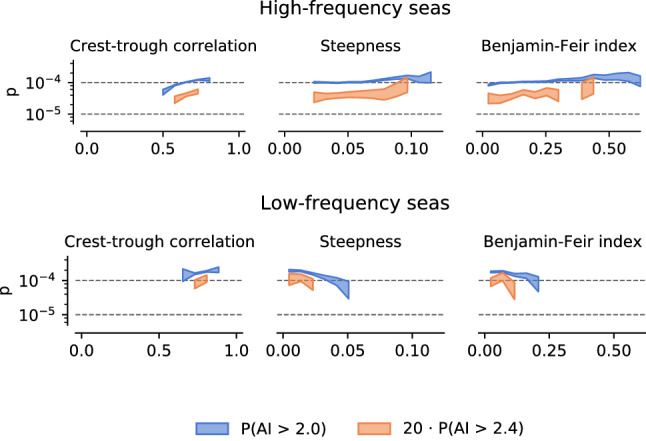
Low-frequency seas have naturally higher rogue wave activity for similar crest-trough correlations, but scale negatively with steepness and BFI. Shown is the scaling of the rogue wave probability *p* with some sea state parameters. Low-frequency/high-frequency conditions are all seas with relative low-frequency energy in the interval (0.8, 0.85) and (0, 0.1), respectively. Curves for $P(\mathrm {AI}> 2.4)$ are scaled by a factor of 20.

This reveals a fundamental difference between these regimes (Fig. 4). Low-frequency seas have naturally higher values of *p*, even for similar values of crest-trough correlation. High-frequency seas show a lower baseline *p*, but are able to reach almost the same maximum *p* through an additional dependency on steepness and Benjamin–Feir index (BFI) that is absent in the low-frequency case. In fact, this relationship is inverted in low-frequency seas, where *p* is *lower* for higher steepness and BFI.

To understand this, it is important to keep in mind that steepness acts on extreme waves in multiple ways. On one hand, steepness is the key parameter in weakly nonlinear modifications to the wave height distribution^17^. On the other hand, steepness also governs wave breaking, an effect that tends to *remove* tall waves^35,36^. Depending on the physical regime, either effect might take over, and fundamentally change the way steepness influences extreme waves.

High-frequency seas can under certain, rare conditions reach about the same rogue wave probabilities as low-frequency seas. The strongest multivariate cluster has a lower bound *p* of $1.6 \cdot 10^{-4}$ for $\mathrm {AI}= 2$ (Supplementary Figure S3). Therefore, the chance to encounter a rogue wave *within a certain time window* is greatest under these conditions (so far, we have only considered the probability *per wave*).

### Rogue crests are governed by skewness, steepness, and Ursell number

Crest heights differ in some fundamental ways from wave heights, since they are affected by second-order nonlinearities that cancel out for wave heights^22^, and they are (by definition) *not* affected by crest-trough correlation. Therefore, we re-run our full analysis for rogue crests.

**Figure 5:**
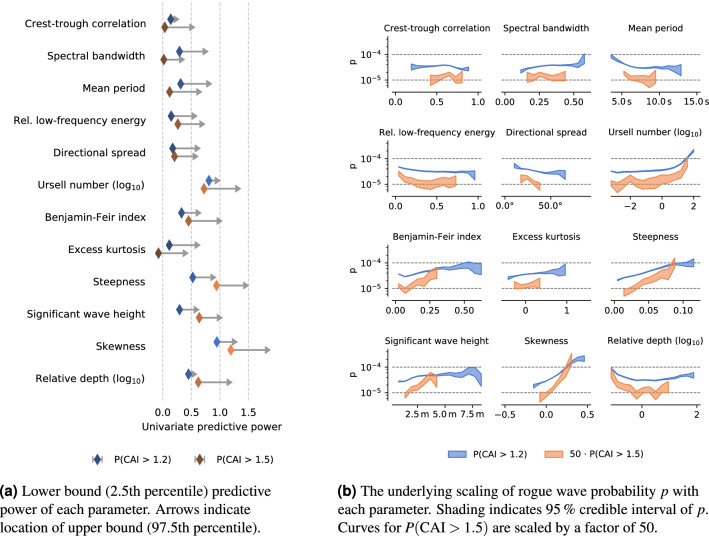
For rogue crests, skewness, steepness, and Ursell number are the most informative parameters. Plots are identical to Fig. 1, except that they refer to crest instead of wave heights.

We find that crest-trough correlation and spectral bandwidth are indeed of very low predictive power (Fig. 5). Instead, surface elevation skewness, steepness, and Ursell number are the strongest parameters, with predictive powers between 0.5 and 1.0. Our multivariate analysis fails to reveal any regions with higher rogue wave probability than the most extreme univariate bin (where $\log _{10}(\text {Ursell number}) \in (1.8, 2.2)$).

A positive skewness indicates steeper crests and flatter, more rounded troughs, and is frequently cited as a proxy for second-order bound nonlinear corrections^13,30,37^. Steepness and Ursell number are the central parameters of the Forristall crest height distribution^25^. Therefore, it seems that rogue crest heights are well explained by second-order theory at this level of detail, but further corrections of up to fourth order may be needed for extremely rare rogue crests^17^.

## Discussion

The results presented during the previous sections are robust to analysis parameter choices and sample size effects (all statements are based on 95% credible intervals).

In particular, we find that our results are stable with regard to sensor location and water depth. To investigate this, we re-ran the analysis on several subsets of the full data, grouped by geographic region (Southern California, Hawaii, US East Coast, West Pacific), relative water depth, and single stations. We did not detect any notable deviations from the dependencies of *p* on the sea state presented above (wherever such comparisons were possible due to the reduced amount of data).

This is surprising, as shallow-water effects and interactions with bathymetry are one hypothesized cause of rogue waves^38,39^. On the other hand, these effects typically require special topographic conditions, which might simply not be present in our data.

The weak dependence of *p* on significant wave height seems to imply that large rogue waves tend to be governed by the same dynamics as small rogue waves. As with location and water depth, we investigated this to some degree by re-running the analysis using only conditions with a significant wave height $> {4} \; \text{m}$. Again, we did not observe notably different scalings of *p* with the sea state (where there were enough data).

We identified crest-trough correlation as the most important parameter for rogue wave formation. It is well understood that bandwidth effects are an important parameter for wave heights^11,21,22^. Perhaps more surprising is the absence of a strong dependency on steepness and BFI, which are central ingredients in current rogue wave prediction^26^, even though we did detect a small positive influence in high-frequency seas (such as storms). For more discussion on the implications of these findings see “Conclusion” section.

Regarding the reliability of our results, some caveats still apply. As the underlying data are supplied by buoys in mostly coastal regions, there are some considerations that might limit the applicability of these results.

Wave buoys are known to underestimate extreme crests through several mechanisms, such as lateral movements around the crest, being dragged through the crest, or linearization of the sea state due to their Lagrangian motion. Even though these effects were found to be of minor importance^40^, we cannot rule out that our conclusions are potentially biased by this. Therefore, our buoy data could underestimate the total number of rogue waves to some degree, and the influence of second-order effects on wave crests might be even higher if measured by a different sensor (this does not affect wave heights, though).

The location of the buoys is another biasing factor. Overall, we are confident that our findings are robust in the studied regime of coastal and island regions in shallow and deep water at moderate significant wave heights, but they might be different in other regions and conditions where we did not have data.

We also do not include any parameters that are not measurable from the sea surface elevation, such as atmospheric conditions (winds), ocean currents, or local topography. There is good evidence that these factors can be important in certain situations^39,41,42^, but since they depend on localized features we do not expect them to be very good predictors in aggregated data from different locations (with the possible exception of winds).

Overall, it is important to keep in mind that our results relate to the rogue wave probability per wave at one given location in space. For extended periods of time and large objects such as oceangoing vessels, the total risk to encounter a rogue wave will be dramatically higher than the probabilities we present here.

## Conclusion

By analyzing over 1 billion wave measurements from buoys, we find that the by far most important parameter for rogue wave occurrence is crest-trough correlation (parameter *r* in the Tayfun distribution^22^). This suggests that, in most conditions, the Rayleigh distribution for Gaussian seas^43^ is in fact an upper bound for real-world rogue waves, as the Tayfun distribution converges to the Rayleigh distribution for $r \rightarrow 1$. Characteristic steepness, BFI, and swell strength provide minor corrections to this. On the other hand, sea surface elevation kurtosis, which is taken as an important indicator for rogue wave activity in many studies^13,29,31^, appears to have no detectable predictive quality when controlling for the fact that rogue waves naturally cause higher kurtosis.

We interpret this as evidence that almost all “freaks” are actually rare realizations of linear or weakly nonlinear seas that are fairly well described by available wave height statistics^22,25^. A similar conclusion has been reached by other, simulation-based studies^13,17^.

This implies that the term *rogue wave* should perhaps be reserved for waves that are truly a “different breed” (such as those caused by modulational instability and other nonlinear effects, or those occurring during a storm), not just any wave that exceeds an arbitrary abnormality index threshold.

Rogue crests seem to be reasonably well-described by second-order, weakly nonlinear theory^25,30,37^, as we found the most important parameters to be skewness, steepness, and Ursell number. However, we did focus on waves during our analysis, so there might be more to uncover—e.g., when conditioning on skewness or different depth regimes.

We also see this work as a demonstration how machine learning methods can be helpful in extreme wave research. Some previous studies have attempted to perform binary classification on rogue wave data^44,45^ (i.e., to predict whether a rogue wave will occur in some block of data or not). We believe that due to the inherently stochastic nature of ocean waves, predicting *rogue wave probabilities* is a better way forward, and have demonstrated that this can lead to tangible insights.

Finally, our statistical and machine learning-based analysis in this study has been purely descriptive. We believe that this work also has important implications for rogue wave *prediction*. Crest-trough correlation can be computed from the wave spectrum, which is routinely forecast globally by agencies like ECMWF. This provides a strong baseline for a rogue wave risk forecast. Combined with more sophisticated machine learning algorithms that are not piecewise constant and take the actual wave height into account (not just binary classification), we are confident that wave height distribution tails will become much more forecastable in the future.

## Methods

### Parameter estimation

Most parameters are taken directly from FOWD without modification. The only exceptions are peak relative depth, Ursell number, and dominant directional spread, which are not part of FOWD, but can be computed based on other FOWD parameters.

An overview over how each parameter is estimated is shown in Table 2. All parameters are based on a 30 min aggregation window.

Since the crest-trough correlation *r* is a central parameter to this article, we give the full expression here^19,22^:

1 $$\begin{aligned} r = \frac{1}{m_0} \sqrt{\rho ^2 + \lambda ^2} \quad \text {with} \quad \rho = \int _0^\infty {S}(\omega ) \cos \bigg (\omega \frac{\overline{T}}{2}\bigg ) \;\mathrm {d}\omega , \quad \lambda = \int _0^\infty {S}(\omega ) \sin \bigg (\omega \frac{\overline{T}}{2}\bigg ) \;\mathrm {d}\omega \end{aligned}$$

where $S(\omega )$ is the wave spectral density, $m_n$ its n-th moment, $\omega$ the angular frequency, and $\overline{T} = m_0 / m_1$ the spectral mean period.

**Table 2:** Overview of how each sea state parameter is estimated from the sea surface elevation.

Parameter	Related FOWD variable(s)	Estimation
Crest-trough correlation	sea_state_30m_crest_trough_correlation	See (1). This represents the envelope of the autocorrelation function at time lag of 1/2 mean zero-crossing period for linear waves
Spectral bandwidth	sea_state_30m_bandwidth_peakedness	Peakedness (quality factor) of wave spectral density^46^
Mean period	sea_state_30m_mean_period_spectral	$\sqrt{m_0/m_2}$, with n-th moment of wave spectral density $m_n$
Rel. low-frequency energy	sea_state_30m_rel_energy_in_frequency_interval	$m_0^{-1} \int S(f) \, \mathrm {d}f$ in the frequency interval 0.05 Hz to 0.1 Hz, with wave spectral density *S*(*f*)
Directional spread	direction_dominant_spread_in_frequency_interval, sea_state_30m_rel_energy_in_frequency_interval	Average over frequency-dependent directional spread weighted with energy in each frequency band
Ursell number ($\log _{10}$)	sea_state_30m_steepness	Ursell number $U = \epsilon / {\tilde{D}}^3$, with relative water depth ${\tilde{D}}$ and characteristic steepness $\epsilon$
Benjamin–Feir index	sea_state_30m_benjamin_feir_index_peakedness	Through characteristic steepness and spectral bandwidth (peakedness)^46^
Excess kurtosis	sea_state_30m_kurtosis	Fourth standardized moment of surface elevation time series
Steepness	sea_state_30m_steepness	Characteristic steepness $\epsilon = \sqrt{2m_0}k_p$ with spectral peak wavenumber $k_p$
Significant wave height	sea_state_30m_significant_wave_height_spectral	Significant wave height $H_S = 4\sqrt{m_0}$
Skewness	sea_state_30m_skewness	Third standardized moment of surface elevation time series
Relative depth ($\log _{10}$)	sea_state_30m_peak_wavelength, meta_water_depth	Relative depth ${\tilde{D}} = D / \lambda _p$ with water depth *D* and peak wavelength $\lambda _p$

### Data preprocessing

We apply the following preprocessing steps to the FOWD wave catalogue:

1. To account for the sampling variability of our relatively low-frequency buoy data, we correct all wave/crest heights and trough depths (and quantities directly derived from them) based on the mean wave period $\overline{T}$ and sampling frequency $f_0$^18^:
  2 $$\begin{aligned} h' = h \cdot \bigg (1 - \frac{\pi ^2}{6 (f_0 \overline{T})^2}\bigg )^{-1} \end{aligned}$$
  As FOWD filtering already removes all records with mean period lower than 5 s for 1.28 Hz CDIP data, this correction factor is quite conservative (maximum possible value of 4.2%).
2. To reduce the 800 GB FOWD-CDIP dataset to a manageable size, we aggregate records into chunks by mapping each 100th sea state to the maximum measured wave height in the upcoming 100 waves.
  This is notably different from the traditional approach to create fixed-*time* chunks (usually 20 min^11,18^). Having a fixed number of waves allows us to directly translate the probability of finding at least one rogue wave within the aggregation window ($p_{100}$) to the rogue wave probability for any given wave (*p*), assuming that all wave heights are identically, independently distributed (*iid.*) within the aggregation period:
  3 $$\begin{aligned} p = 1 - (1 - p_{100})^{{1}/{100}} \end{aligned}$$
  This process also removes the influence of multiple rogue waves occurring back-to-back, because we only measure the probability that at least one wave in the record is a rogue wave. This has an additional regularizing effect that prevents the analysis from over-emphasizing conditions which have a tendency for multiple rogue waves.

All preprocessed data are freely available for download (see data availability statement).

### Univariate binning

In the univariate case, we split all wave height observations into *N* equal-sized bins for each sea state parameter *x*. Our analysis then hinges on the assumption that all binary samples within a bin (consisting of $n^+$ rogue and $n^-$ non-rogue observations) are identically, independently distributed (*iid.*) according to a binomial distribution with rogue wave probability *p* as the only parameter. Our goal is to estimate *p*, which we interpret in Bayesian fashion as a random variable, from measurements of $n^+$ and $n^-$ within each bin (we introduced this process in the initial publication of FOWD^19^).

For *p* we assume a Beta distributed prior with parameters $\alpha _0$, $\beta _0$ (Table 3). The role of this prior is to constrain *p* to a reasonable order of magnitude, while being weakly informative so the exact choice of parameters does not influence final results.

Because the Beta prior is conjugate to the binomial likelihood, we obtain for the posterior of *p*:

4 $$\begin{aligned} P(p \mid n^+, n^-) = {\text {Beta}}(n^+ + \alpha _0,\, n^- + \beta _0) \end{aligned}$$

Since this is just another Beta distribution, the posterior for *p* is easy to evaluate with any modern statistical software. Specifically, we quantify our best estimate for *p* through the median of (4), and our uncertainty by the 95% credible interval (based on quantiles of the posterior).

The assumption that measurements are iid. within each univariate bin is obviously not fulfilled if *p* depends on more than one sea state parameter, so the uncertainties obtained through this process can only give an indication of our confidence in the marginal rogue wave probability when we can only measure one parameter at a time. We also need to pick small enough bins such that the variance of the true *p*(*x*) is small within each bin.

In the case of aggregated data, we model $p_{100}$ instead of *p* via (4), where $n^+$/$n^-$ relate to the number of 100-wave chunks containing a rogue wave/no rogue waves, and with $\beta _0$ reduced by a factor of 100. After estimating the desired statistical properties of $p_{100}$ (median and quantile-based credible interval), we translate those into the corresponding values of *p* via (3) (all reported quantities are *per wave*).

**Table 3:** Beta prior parameters for *p* for different wave ($\mathrm {AI}$) and crest ($\mathrm {CAI}$) height thresholds.

	$\alpha _0$	$\beta _0$
$\mathrm {AI}> 2$	1	10,000
$\mathrm {AI}> 2.4$	1	1,000,000
$\mathrm {CAI}> 1.2$	1	10,000
$\mathrm {CAI}> 1.4$	1	1,000,000

### Predictive power

We define the “predictive power” ${\mathbb {P}}_x$ of a parameter *x* as:

5 $$\begin{aligned} {\mathbb {P}}_x&= \log _{10} \bigg ( \frac{p_{i_\text {max}}}{p_{i_\text {min}}} \bigg ) \end{aligned}$$

6 $$\begin{aligned} i_\text {max}&= \underset{i}{{\text {argmax}}} \big [ Q_{0.025} (p_i) \big ]&\text {(bin index with highest lower bound }p) \end{aligned}$$

7 $$\begin{aligned} i_\text {min}&= \underset{i}{{\text {argmin}}} \big [Q_{0.975} (p_i) \big ]&\text {(bin index with lowest upper bound } p) \end{aligned}$$

where $p_i$ denotes the value of *p* in the *i*-th bin of *x*, and $Q_q (p_i)$ denotes the q-th quantile of $p_i$. This measures how much of the variation of *p* is explained by *x* (if we can only consider this one parameter) in a way that is robust to sample size effects. We also quantify our uncertainty in ${\mathbb {P}}_x$ through Monte Carlo sampling, based on the known distributions of $p_{i_\text {max}}$ and $p_{i_\text {min}}$ as given in (4).

### High-dimensional clustering

To account for interactions between sea state parameters, we use a decision-tree based clustering algorithm to identify rectangular regions in feature space where the rogue wave probability is higher than any probability obtained via univariate analysis.

At its core, the algorithm is a two-step process:

1. Fit a deep random forest classifier to binary data to obtain ${\tilde{p}}(X)$, which is a rough, noisy estimate of *p*(*X*). Here, *X* denotes the vector of *all* sea state parameters *x*.
2. Fit a shallow decision tree regressor to $\log {\tilde{p}}(X)$ (with mean squared error criterion). The leaves of this surrogate model then represent the desired clusters wherein *p*(*X*) is approximately constant. We find and retain the 12 leaves with the highest (significant) imbalance between classes.

As this process represents a model search it is vulnerable to overfitting. Therefore, we only use 34% of all available data to identify clusters, and the remaining 66% of the data to analyze the conditions within the cluster (i.e., they determine the final reported rogue wave probability).

This is a conservative process, where all estimators are piecewise constant, which severely limits their learning capabilities. On the other hand, this process should be robust to overfitting, its outputs are easy to analyze (since they just represent another rectangular bin in feature space), and the efficient computation of decision trees ensures that it can scale to billions of data points.

For the decision tree and random forest algorithms, we used the implementations by scikit-learn^47^. The full implementation of our analysis is available as a Jupyter notebook (see Data availability section) that can be used to reproduce all plots in this publication.

## Supplementary Information

Supplementary Information.

## Data Availability

All preprocessed input data are available at 10.17894/ucph.99bab774-2c97-4e9f-871f-3c349cc0d510. The Jupyter notebook used to generate the results and figures in this report is available at 10.5281/zenodo.4724496.
