# Supplementary material for: Real-world rogue wave probabilities
Source: Sci Rep. 2021 May 12;11:10084. doi: 10.1038/s41598-021-89359-1 (PMC8115049; doi:10.1038/s41598-021-89359-1)
Supplement: Supplementary file 1 — Supplementary Information. [file 41598_2021_89359_MOESM1_ESM.pdf]

Real-world Rogue Wave Probabilities — Supplementary Information

Dion Häfner[1,*], Johannes Gemmrich[2], and Markus Jochum[1]

[1]Niels Bohr Institute, University of Copenhagen, Copenhagen, Denmark
[2]University of Victoria, Victoria, British Columbia, Canada
[*]dion.haefner@nbi.ku.dk

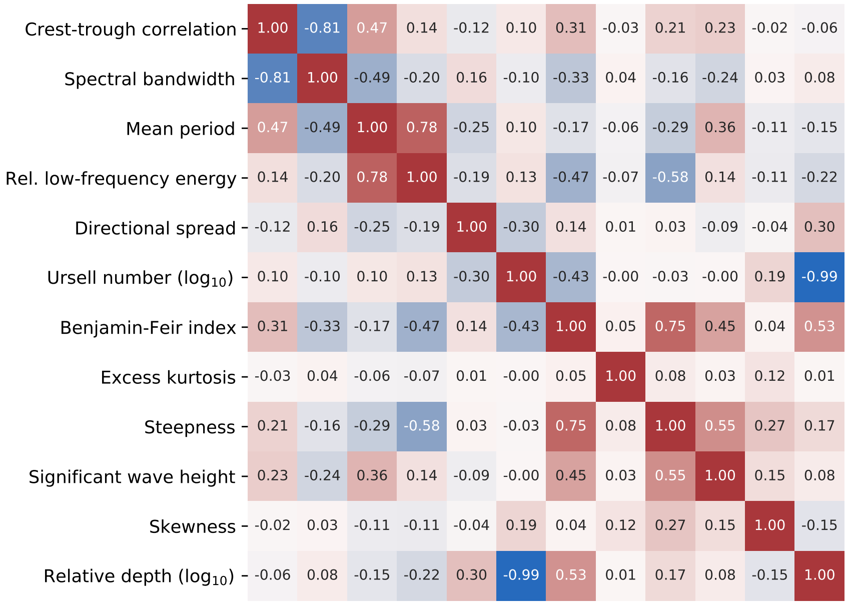

Supplementary Figure S1. Linear (Pearson) correlation matrix between sea state parameters.

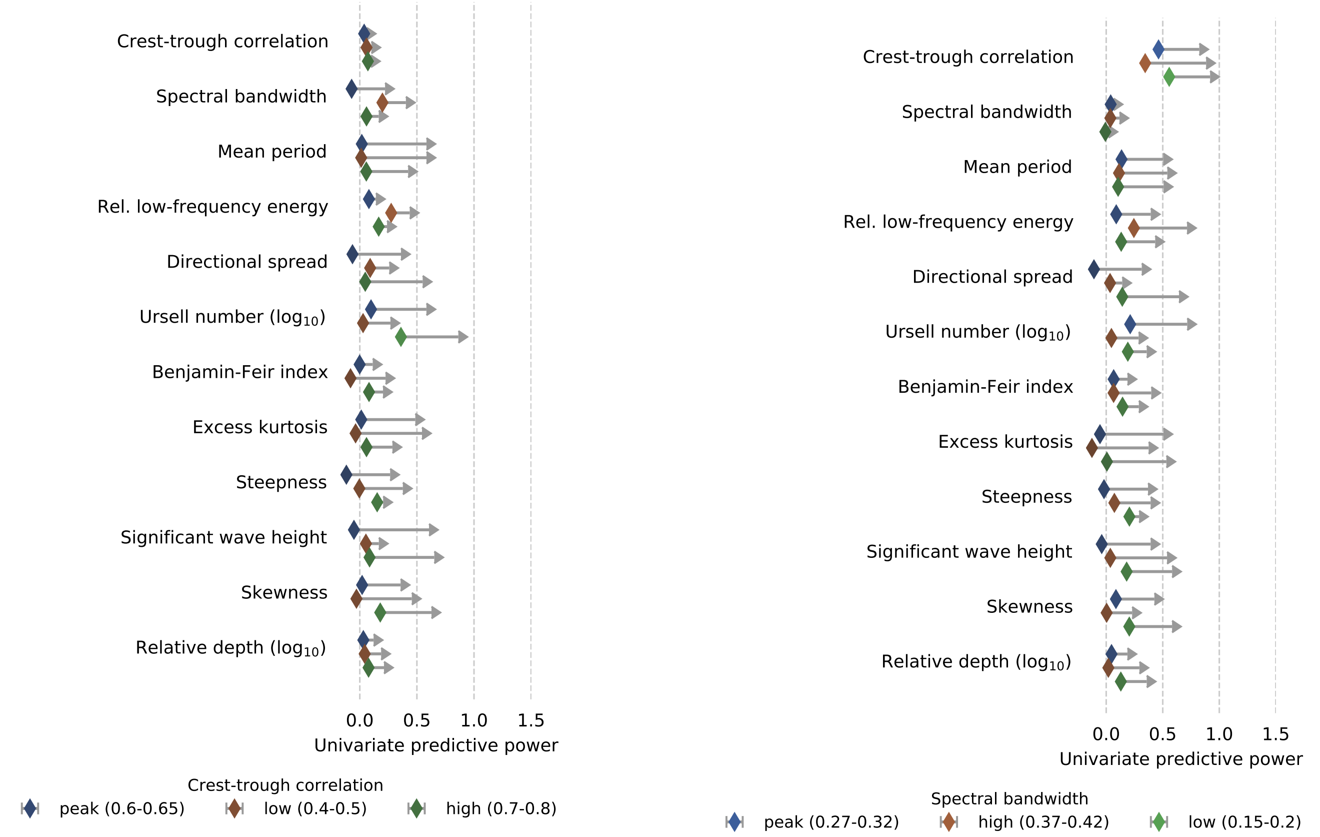

(a) Predictive power when stratifying on crest-trough correlation.

(b) Predictive power when stratifying on spectral bandwidth.

Supplementary Figure S2. When stratifying on crest-trough correlation, spectral bandwidth loses its predictive power, but not vice-versa. Plots are analogous to Figure 1a in the main article.

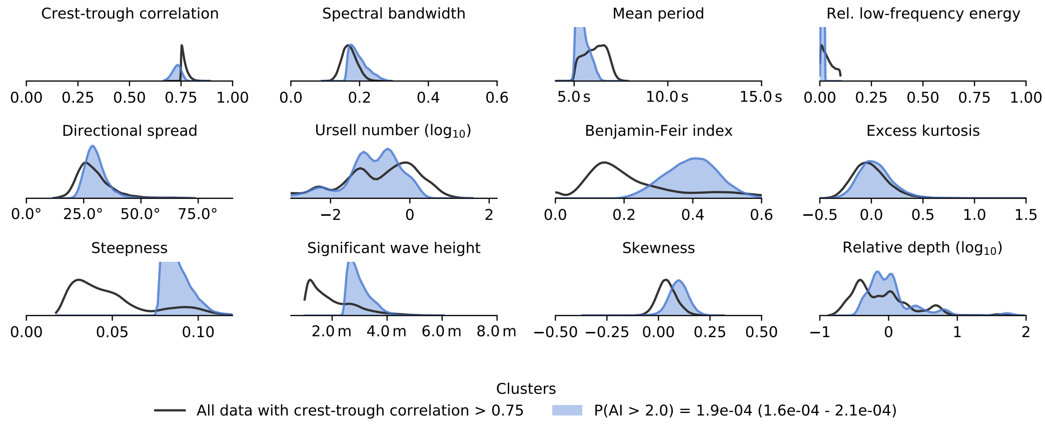

Supplementary Figure S3. Under rare conditions, there can be almost as many rogue waves in high-frequency seas as in low-frequency seas. Shown is the parameter distribution of the most extreme cluster in high-frequency seas (i.e., seas with less than 10 % total energy in the 0.05 Hz to 0.1 Hz frequency band). Rogue wave probability ranges in legend indicate 95 % credible interval. Plot is analogous to Figure 2 in the main article.
